# Geranylgeranylacetone attenuates cerebral ischemia–reperfusion injury in rats through the augmentation of HSP 27 phosphorylation: a preliminary study

**DOI:** 10.1186/s12868-021-00614-7

**Published:** 2021-02-08

**Authors:** Kazuya Matsuo, Kohkichi Hosoda, Jun Tanaka, Yusuke Yamamoto, Taichiro Imahori, Tomoaki Nakai, Yasuhiro Irino, Masakazu Shinohara, Takashi Sasayama, Eiji Kohmura

**Affiliations:** 1 Department of Neurosurgery, Kobe University Graduate School of Medicine, Kobe, Japan; 2 Department of Neurosurgery, Kobe City Nishi-Kobe Medical Center, 5-7-1, Kojidai, Nishi-ku, Kobe, Hyogo 651-2273 Japan; 3 Department of Neurosurgery, Konan Hospital, Kobe, Japan; 4 Department of Neurosurgery, Toyooka Hospital, Toyooka, Japan; 5 Department of Neurosurgery, Hyogo Brain and Heart Center at Himeji, Himeji, Japan; 6 Division of Evidence-based Laboratory Medicine, Kobe University Graduate School of Medicine, Kobe, Japan; 7 Division of Medical Education, Kobe University Graduate School of Medicine, Kobe, Japan

**Keywords:** Glucose-6-phosphate dehydrogenase, Heat shock protein, Metabolomics, Oxidative stress, Reperfusion injury, Stroke

## Abstract

**Background:**

We previously reported that heat shock protein 27 (HSP27) phosphorylation plays an important role in the activation of glucose-6-phosphate dehydrogenase (G6PD), resulting in the upregulation of the pentose phosphate pathway and antioxidant effects against cerebral ischemia–reperfusion injury. The present study investigated the effect of geranylgeranylacetone, an inducer of HSP27, on ischemia–reperfusion injury in male rats as a preliminary study to see if further research of the effects of geranylgeranylacetone on the ischemic stroke was warranted.

**Methods:**

In all experiments, male Wistar rats were used. First, we conducted pathway activity profiling based on a gas chromatography–mass spectrometry to identify ischemia–reperfusion-related metabolic pathways. Next, we investigated the effects of geranylgeranylacetone on the pentose phosphate pathway and ischemia–reperfusion injury by real-time polymerase chain reaction (RT-PCR), immunoblotting, and G6PD activity, protein carbonylation and infarct volume analysis. Geranylgeranylacetone or vehicle was injected intracerebroventricularly 3 h prior to middle cerebral artery occlusion or sham operation.

**Results:**

Pathway activity profiling demonstrated that changes in the metabolic state depended on reperfusion time and that the pentose phosphate pathway and taurine-hypotaurine metabolism pathway were the most strongly related to reperfusion among 137 metabolic pathways. RT-PCR demonstrated that geranylgeranylacetone did not significantly affect the increase in HSP27 transcript levels after ischemia–reperfusion. Immunoblotting showed that geranylgeranylacetone did not significantly affect the elevation of HSP27 protein levels. However, geranylgeranylacetone significantly increase the elevation of phosphorylation of HSP27 after ischemia–reperfusion. In addition, geranylgeranylacetone significantly affected the increase in G6PD activity, and reduced the increase in protein carbonylation after ischemia–reperfusion. Accordingly, geranylgeranylacetone significantly reduced the infarct size (median 31.3% vs 19.9%, p = 0.0013).

**Conclusions:**

As a preliminary study, these findings suggest that geranylgeranylacetone may be a promising agent for the treatment of ischemic stroke and would be worthy of further study. Further studies are required to clearly delineate the mechanism of geranylgeranylacetone-induced HSP27 phosphorylation in antioxidant effects, which may guide the development of new approaches for minimizing the impact of cerebral ischemia–reperfusion injury.

## Background

Ischemic stroke is the leading cause of morbidity and mortality, although recent developments in endovascular thrombectomy for acute ischemic stroke have dramatically improved patient outcomes [1]. After ischemia, there are two different types of injury: primary anoxic ischemic cell death and delayed secondary neuronal injury induced by postischemic reperfusion [2]. Such ischemia–reperfusion injury is an important therapeutic target because the incidence of ischemia–reperfusion injury will continue to increase in accordance with the increase in successful mechanical reperfusion with thrombectomy. Ischemia–reperfusion injury is characterized by a cytotoxic cascade with an excess of reactive oxygen species (ROS) and the hyperactivation of inflammatory cytokines and nitric oxide, leading to neuronal cell death, redox imbalance, inflammation, and apoptosis [2]. Consequently, ischemia–reperfusion injury can increase the infarct size and worsen blood brain barrier breakdown, leading to brain edema and hemorrhage and resulting in disastrous outcomes [3]. Therefore, it is crucial to protect the brain from secondary injury caused by reperfusion for a better outcome. There is currently no effective treatment for cerebral ischemia–reperfusion injury, although many studies have attempted to develop new therapeutic agents for ischemia–reperfusion injury [4].

Previously, we reported the importance of pentose phosphate pathway (PPP) activation in primary ischemic injury based on the results of a metabolomics analysis of the rat cerebral cortex [5]. In particular, we found that heat shock protein (HSP) 27 phosphorylation plays an important role in the activation of glucose-6-phosphate dehydrogenase (G6PD), the rate-limiting enzyme of the PPP, resulting in the upregulation of the PPP and antioxidant effects upon reduced nicotinamide adenine dinucleotide phosphate (NADPH) synthesis. Furthermore, we detected that this endogenous antioxidant system with the upregulation of PPP is also activated during postischemic reperfusion [6]. Accordingly, the induction of HSP27 or HSP27 phosphorylation seems to promote the PPP, the endogenous antioxidant system, and attenuates cerebral ischemia–reperfusion injury.

In this study, we first conducted a metabolomics analysis with gas chromatography-mass spectrometry (GC–MS) to determine reperfusion-related metabolic pathways. Second, as a preliminary investigation to assess the efficacy of the geranylgeranylacetone (GGA), which is an inducer of HSP including HSP27 [7–9], we performed preischemic intracerebroventricular injection of GGA to overexpress the HSP27 and evaluated its effect on cerebral ischemia–reperfusion injury in rats.

## Results

### Metabolic changes in the cerebral cortex after ischemia–reperfusion

**Fig. 1:**
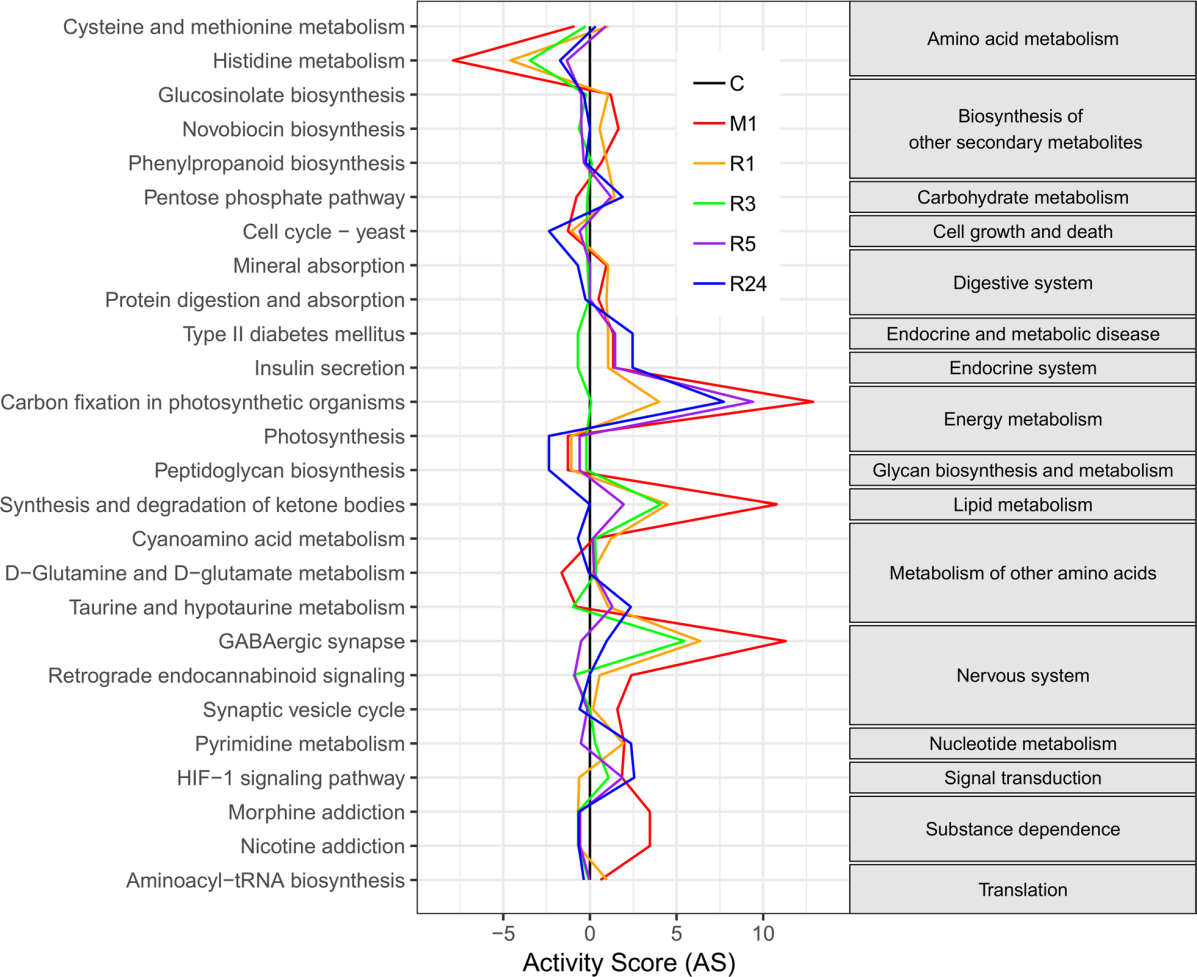
Pathway activity profiling graph. The activity score for each metabolic pathway was plotted using the control (sham-operated) group as a reference. The activity score were based on 96 metabolites identified by gas chromatography–mass spectrometry analysis of cerebral cortex derived from rats from the middle cerebral artery occlusion (MCAO)-induced ischemia followed by some duration of reperfusion (1, 3, 5, 24-h) groups and the control group. Only pathways with statistical significance (P < 0.05) are shown. The pentose phosphate pathway was the highly ranked pathway, and the pattern of activity score in the pentose phosphate pathway was unique (see text for details). The right column demonstrates Kyoto Encyclopedia of Genes and Genomes orthology. C: control group; M1: 1-h MCAO without reperfusion group; R1: 1-h reperfusion after 1-h MCAO group; R3: 3-h reperfusion after 1-h MCAO group; R5: 5-h reperfusion after 1-h MCAO group; R24: 24-h reperfusion after 1-h MCAO group

To obtain detailed information on changes in the metabolic profile in the cerebral cortex during ischemia–reperfusion, pathway activity profiling (PAPi) analysis was performed by using GC–MS results. Four rats were excluded from the GC–MS analysis due to incomplete regional cerebral blood flow (rCBF) restoration after thread removal. Three were excluded due to incomplete rCBF reduction during surgery. Three were excluded because of subarachnoid hemorrhage, and three died within reperfusion period. The pre-processed data of GC–MS results were shown in Additional file 1: Table S1. PAPi searches each of the 137 metabolic pathways in the Kyoto Encyclopedia of Genes and Genomes (KEGG) database. According to the criterion of p < 0.05 with Bonferroni correction, the results clearly demonstrated significant differential regulation of 26 metabolic pathways in the rat cerebral cortex during ischemia–reperfusion. These were associated with amino acid metabolism, carbohydrate metabolism, energy metabolism, lipid metabolism, other amino acid metabolism and nucleotide metabolism (Fig. 1). In the PAPi analysis, the PPP was a highly ranked pathway, which is in accordance with the results of our previous studies [5, 6]. According to KEGG, metabolites such as Ribulose-5-phosphate and Fructose-6-Phosphate are involved in PPP. Furthermore, the activity score of the PPP and taurine/hypotaurine metabolism were low in the ischemia with no reperfusion group but high in the ischemia with some duration of reperfusion groups. This trend was not observed for the other metabolic pathways.

### Effect of GGA on the expression patterns of HSP27 and G6PD during ischemia–reperfusion

The results of PAPi analysis suggested that the significant differential regulation of the PPP occurred during cerebral ischemia–reperfusion. G6PD is the rate-limiting enzyme of the PPP and reduces NADP to NADPH, which is then utilized by glutathione reductase to reduce glutathione disulfide (GSSG), leading to antioxidant effects. Our previous work demonstrated that HSP27 is upregulated during ischemia–reperfusion, which has been reported to increase the activity of G6PD [10]. Thus, we focused our subsequent investigation on the effect of inducing HSP27 by GGA during cerebral ischemia–reperfusion.

**Fig. 2:**
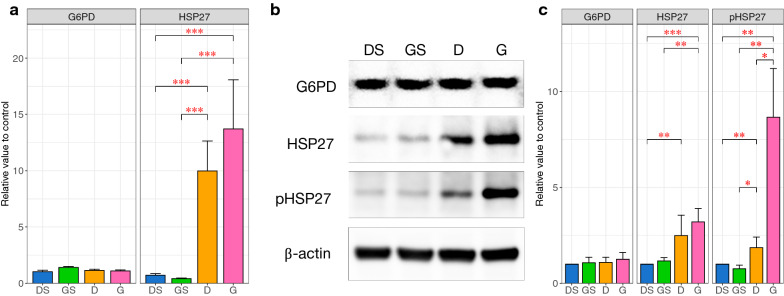
Effect of the intracerebroventricular geranylgeranylacetone injection on HSP27, pHSP27 and G6PD. **a** Quantitative real-time polymerase chain reaction analysis of rat cerebral cortex after ischemia–reperfusion. No statistically significant difference was observed in the heat shock protein (HSP) 27 mRNA level between groups D and G. **b** Immunoblotting analysis of rat cerebral cortex after ischemia–reperfusion using the indicated antibodies. The glucose 6-phosphate dehydrogenase (G6PD), HSP27, phosphorylated HSP27, and β-actin immunoblots shown were prepared from the same gel. The cropped blots are used in the figure. Full-length blots are presented in Additional files 2: Figure S1, 3: Figure S2, 4: Figure S3, 5: Figure S4. **c** The relative expression levels of the proteins were determined through densitometric evaluation of the immunoblots and were normalized to the expression level of β-actin. No significant difference in HSP27 expression was observed between groups D and G, but the pHSP27 protein level was significantly higher in group G than in group D. The columns represent the average of each group. The error bars are standard error of mean. A nonparametric Tukey–Kramer test was performed to compare each experimental mean (*P < 0.05; **P < 0.01; ***P < 0.001). DS: dimethylsulfoxide injection followed by sham operation group (control); GS: geranylgeranylacetone (GGA) injection followed by sham operation group; D: dimethylsulfoxide injection followed by middle cerebral artery occlusion (MCAO) and reperfusion group; G: GGA injection followed by MCAO and reperfusion group; G6PD, glucose 6-phosphate dehydrogenase; HSP: heat shock protein; pHSP: phosphorylated heat shock protein

In the following experiments, Four rats were excluded from analysis due to incomplete rCBF reduction during surgery. Two rats were excluded due to incomplete rCBF restoration after thread removal. One was excluded because of subarachnoid hemorrhage, and one died during surgery. The expression of HSP27 and G6PD mRNA was measured by quantitative real-time polymerase chain reaction (RT-PCR). HSP27 mRNA levels were significantly increased by 14-fold in group D (p < 0.0001) and by 19-fold in group G (p < 0.0001) compared with the controls. There was no statistically significant difference in HSP27 mRNA levels between groups D and G. In contrast, G6PD mRNA levels did not change during ischemia–reperfusion with or without GGA administration (Fig. 2a).

Immunoblot densitometry results showed no significant changes in G6PD protein levels during ischemia–reperfusion with or without GGA administration. In contrast, after 24-h reperfusion following 1-h middle cerebral artery occlusion (MCAO), the HSP27 protein level was significantly elevated by 2.5-fold in group D (p = 0.0048) and by 3.2-fold in group G (p = 0.00064) compared with the controls. No significant difference in HSP27 protein levels was observed between groups D and G. On the other hand, the phosphorylation of HSP27 at serine 85 was significantly elevated by 1.9-fold in group D (p = 0.0026) and 8.7-fold in group G (p = 0.0013) compared with the controls. The pHSP27 protein levels in group G were significantly higher than those in group D (p = 0.025) (Fig. 2b, and Additional files 2: Figure S1, 3: Figure S2, 4: Figure S3, 5: Figure S4), which means that the phosphorylation of HSP27 was induced more strongly by GGA than by ischemia–reperfusion alone.

### Effect of GGA on G6PD activity and protein carbonyl levels in ischemia–reperfusion injury

**Fig. 3:**
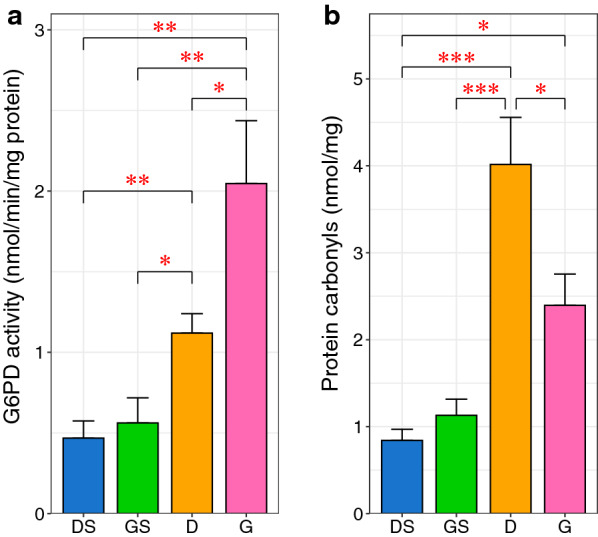
Effect of the intracerebroventricular geranylgeranylacetone injection on glucose 6-phosphate dehydrogenase activity and protein carbonyl levels. **a** glucose 6-phosphate dehydrogenase (G6PD) activity in group G was significantly higher than that in group D. **b** The increase in protein carbonyl levels was significantly inhibited by geranylgeranylacetone (GGA). The protein carbonyl levels are used as an indicator of oxidative stress. The columns represent the average of each group. The error bars are standard error of mean. A nonparametric Tukey–Kramer test was performed to compare each experimental mean (*P < 0.05; **P < 0.01; ***P < 0.001). DS: dimethylsulfoxide injection followed by sham operation group (control); GS: GGA injection followed by sham operation group; D: dimethylsulfoxide injection followed by middle cerebral artery occlusion (MCAO) and reperfusion group; G: GGA injection followed by MCAO and reperfusion group

Although the results of both RT-PCR and immunoblot analysis showed that G6PD levels did not change among the groups, G6PD activity was significantly increased by 2.4-fold in group D (p = 0.0033) and by 4.4-fold in group G (p = 0.0011) compared with the controls. Furthermore, G6PD activity in group G was significantly higher than that in group D (2.05 vs 1.12 nmol/min/mg protein, p = 0.045), which means that G6PD activity was more strongly increased by GGA than by ischemia–reperfusion alone (Fig. 3a).

To evaluate the cerebral ischemia–reperfusion-induced oxidative damage, the generation of protein carbonyl derivatives was monitored. The levels of protein carbonyls were significantly increased by 4.8-fold in group D (p = 0.0002) and by 2.8-fold in group G (p = 0.029) compared with the controls (Fig. 3b). Notably, the protein carbonyl levels were significantly lower in group G than in group D, which means that GGA significantly reduced the protein carbonyl levels during ischemia–reperfusion (p = 0.023).

### Effect of GGA on infarct volume in ischemia–reperfusion injury

**Fig. 4:**
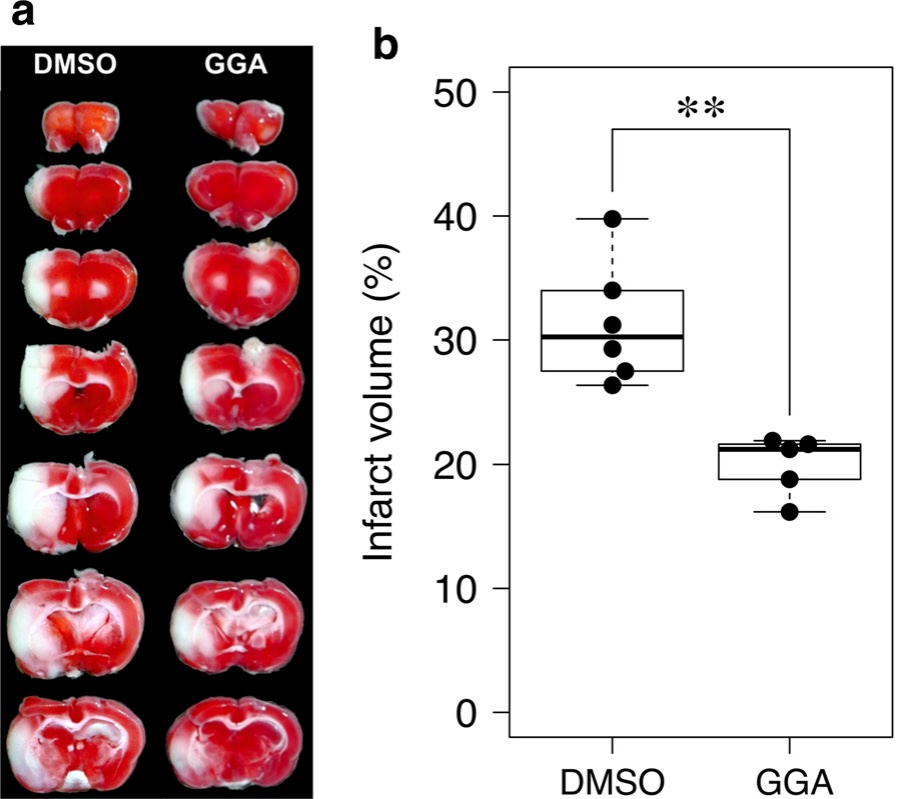
Effect of the intracerebroventricular geranylgeranylacetone injection on the infarct volume after ischemia–reperfusion injury. **a** Typical difference in the infarct volumes induced by 24-h reperfusion after 1-h middle cerebral artery occlusion (MCAO) between the geranylgeranylacetone (GGA)-treated group and the control group. **b** Box-and-whisker plots of the infarct volumes. GGA administration significantly reduces the infarct volume. The thick horizontal lines divide the boxes at the median value. The lower and upper regions of the boxes represent the first and third quartiles, respectively. Each circle represents the value obtained for an individual sample. Mann–Whitney U tests were performed for comparisons (*P < 0.05; **P < 0.01). DMSO: dimethylsulfoxide

Three rats were excluded from the infarct volume analysis due to incomplete rCBF reduction during surgery, and one was excluded because of subarachnoid hemorrhage. 1% 2, 3, 5-Triphenyltetrazolium chloride (TTC) staining of rat brain coronal sections (Fig. 4a) showed that the preischemic administration of GGA significantly reduced the infarct volume from 31.3 ± 4.9% to 19.9 ± 2.4% (p = 0.0013) (Fig. 4b). The reduction of rCBF during MCAO was not significantly different between group G and group D (data not shown).

## Discussion

### PAPi analysis suggests a significant differential change in the PPP during ischemia–reperfusion

PAPi analysis of the GC–MS-based metabolomics data demonstrated significant differential regulation of 26 of the 137 KEGG metabolic pathways in the rat cerebral cortex during ischemia–reperfusion. Similar to our previous works, which demonstrated that the PPP is significantly upregulated during ischemia–reperfusion [6], the current PAPi analysis also confirmed that the PPP is one of the pathways that is significantly differentially regulated during ischemia–reperfusion. The PPP generates NADPH, which serves as a cofactor for glutathione reductase. Glutathione reductase produces GSSG, which acts together with glutathione peroxidase to remove ROS [10]. Therefore, it is likely that the increased PPP activity forms a part of the antioxidant system in the brain during ischemia–reperfusion. Accordingly, we focused the following investigation on the effect of PPP upregulation due to the induction of HSP27 by GGA during cerebral ischemia–reperfusion. The significant differential regulation of other pathways was also observed in the present study. However, these findings are beyond the scope of the current work. Future studies will focus on these pathways.

### GGA

GGA was first reported to enhance the expression of the HSP family in the cultured gastric mucosa [11]. Thereafter, in various animal models of ischemia–reperfusion, GGA shows a protective effect against oxidative stress in various organs, including the liver [12], heart [13], and kidney [14]. However, there have been very few reports regarding the effect of GGA in cerebral ischemia–reperfusion injury [15, 16]. In addition, these studies did not fully examine the mechanism by which GGA provides neuroprotection against ischemia–reperfusion injury and did not focus on HSP27 and/or pHSP27. In the current study, GGA was employed to induce HSP27 in the rat cerebral cortex to assess the effect of PPP upregulation on ischemia–reperfusion injury. The current study demonstrated that GGA can protect the rat cerebral cortex against ischemia–reperfusion injury by increasing the endogenous phosphorylation of HSP27, which activates G6PD activity, during ischemia–reperfusion. However, the present research is only a preliminary study that needs further evaluation of optimal therapeutic window and effective administration route.

### Transcriptional analysis confirms that the upregulation of HSP27 by cerebral ischemia–reperfusion is not influenced by GGA

Our previous study demonstrated that ischemia–reperfusion induces the upregulation of HSP27 transcription [6]. The current RT-PCR results confirmed that GGA does not affect the steady-state transcription level of HSP27 in the brain. Furthermore, GGA does not significantly affect the upregulation of HSP27 transcription by ischemia–reperfusion. Although the induction of HSP27 by GGA has been reported in various organs, including the heart [9], liver [8], and cochlear nerve [7], there have been no studies showing HSP27 induction in the brain by GGA.

### GGA enhances G6PD activation via an increase in HSP27 phosphorylation and reduces the infarct volume during ischemia–reperfusion

Our previous study demonstrated a significant increase in HSP27 protein levels after 24 h reperfusion following 1 h of MCAO [6]. The current immunoblotting results confirmed that GGA does not affect the steady-state protein level of HSP27 in the brain in the absence of ischemia–reperfusion. In addition, GGA does not significantly affect the ischemia–reperfusion-induced elevation of HSP27 protein levels. However, GGA significantly enhances the endogenous increase in HSP27 phosphorylation induced by ischemia–reperfusion although GGA does not affect the steady-state phosphorylation level of HSP27. This implies that GGA increases HSP27 phosphorylation only during ischemia–reperfusion. Similar results showing that GGA induces the overexpression of HSPs under stress conditions but has a weak induction effect in the absence of stress have been reported [13, 17].

It has been reported that the interaction of G6PD with highly phosphorylated small oligomers of HSP27 results in increased G6PD activity [18]. The phosphorylation of HSP27 at serine 15 and serine 82 by protein kinase D has also been demonstrated to be decisive for HSP27-induced neuroprotection against ischemic neuronal injury in a mouse ischemia–reperfusion model [19]. Serine 82 in human HSP27 corresponds to serine 85 in rat HSP27; the latter is the specific site that we identified as being phosphorylated during ischemia–reperfusion and that we evaluated in the current study.

Since metabolic pathways are primarily modulated by posttranslational mechanisms [20], available information concerning transcription and translation levels is limited; thus, an accurate assessment of changes in PPP activity are hampered. Therefore, it is necessary to determine mRNA and protein levels along with enzyme activity. The current study demonstrated that GGA significantly enhances the endogenous increase in G6PD activity induced by ischemia–reperfusion.

The results of the current study demonstrated that GGA significantly enhances the endogenous increase in G6PD activity induced by ischemia–reperfusion. Consistent with the increase in G6PD activity, the current study demonstrated that GGA significantly reduces the elevation of protein carbonyl content and reduces the infarct volume after 24-h reperfusion following 1-h MCAO. G6PD in the PPP is the main supplier of NADPH [21], which confers protection against ROS. Accordingly, it seems reasonable that the enhancement of G6PD activity through HSP27 phosphorylation induced by GGA attenuates protein carbonylation and the severity of cerebral infarct. A previous report evaluated the effect of GGA on infarct size in a permanent ischemic model rather than a cerebral ischemia–reperfusion model [17]. The authors demonstrated the protective effect of GGA in a mouse model of permanent MCAO. GGA (1000 mg/kg) was injected intraperitoneally 1 h prior to the onset of ischemia. The infarct volume in the treated groups was significantly reduced compared with that in the controls. However, GGA did not reduce the infarct volume when administered at 1 h after the onset. Further studies are required to determine the optimal therapeutic window of GGA administration for neuroprotection against cerebral ischemia–reperfusion injury.

### Protective effect of HSP27 phosphorylation against ischemia–reperfusion injury

A recent study demonstrated that the overexpression of HSP27 provides cellular protection against cerebral ischemia [22–26]. Although these previous studies demonstrated the neuroprotective effect of HSP27 against stroke, all of these studies induced HSP27 by using a viral vector or transgenic overexpression. Another recent study reported that an intravenous injection of an in vitro-phosphorylated recombinant HSP27 reduces the infarct size and improves neurological deficits in mice [27]. Further studies are necessary to establish a feasible and practical approach for the induction of HSP27 and/or pHSP27 overexpression for the treatment of stroke. The present study was the first to demonstrate that GGA induces the phosphorylation of HSP27 and activates G6PD, leading to an antioxidant effect against ischemia–reperfusion injury. The effects of GGA on neurological outcome should be further investigated in future studies.

Recent trials of mechanical thrombectomy have shown a dramatic improvement in the outcomes of patients with acute stroke [1]. However, the benefit was found to decrease as the interval between the time at which the patient was last known to be well and thrombectomy increased. Therefore, the activation of G6PD via the enhancement of HSP27 phosphorylation induced by GGA may be used to widen the therapeutic time window and to protect neural tissue against reperfusion injury, which may improve the results of stroke treatment with mechanical thrombectomy. Further studies are required prior to clinical application of GGA, evaluating the effect of GGA administration after MCAO. In this study, we used pre-ischemic administration of GGA to study different pathways or biological processes.

### Limitation

There are several limitations to this study. First, we could not exclude the influence of edema on the calculation of the infarct volume because cerebroventricular puncture made measuring the volume of the contralateral hemisphere difficult [28]. For example, the contralateral contusion made by cerebroventricular puncture was not stained by TTC as shown in Fig. 4. Further investigation of a more convenient administration route, such as intraarterial or intravenous, is needed. Although the geranylgeranylacetone can be administered orally [29], it was unclear whether the effect of oral administration was sufficient. Therefore, we used intraventricular injection to ensure its effect as a preliminary study. Second, GGA also induces the expression of several stress-related proteins, such as HSP70 [12–14, 17], HSP90 [16], HSPB8 [9], and endothelial nitric oxide synthase [16]. Thus, the protective effect of GGA observed in the current study cannot be explained solely by the effect of HSP27 and its phosphorylation. The role of other protective proteins and their potential association with oxidative stress requires further study. Third, PAPi searches every metabolic pathway in the KEGG database. Therefore, it may report metabolic pathways not necessarily related to the organism under study, such as cell cycle-yeast and photosynthesis.

## Conclusions

The current data indicate that the pre-ischemic intracerebroventricular administration of GGA reduces the infarct volume and oxidative stress caused by cerebral ischemia–reperfusion. The mechanism of this effect was through the enhancement of G6PD activity due to HSP27 phosphorylation induced by GGA. These findings suggest that GGA may be a promising agent for the treatment of ischemic stroke. However, the present study is only a preliminary study that should be complemented by further research on optimal therapeutic window and effective administration route based on long-term follow-up and neurological evaluation. Further studies are also required to clearly delineate the mechanism by which GGA-induced HSP27 phosphorylation exerts antioxidant effects, which may guide the development of new approaches to minimize the impact of cerebral ischemia–reperfusion injury.

## Methods

### Animals and drugs

Male Wistar rats, aged 8–10 weeks weighing 220–260 g (Japan SLC), were maintained at 22 ± 2 °C under a 12-h light/dark cycle with free access to food and water. The clinical signs of rats were observed at least twice a day. If the criteria of humane endpoints were met, rats were sacrificed. Humane endpoints were considered as severe respiratory distress, poor physical appearance, seizure, vomiting or skin problems (wounds or signs of inflammation). The animal experiments were performed under a protocol approved by the Institutional Animal Care and Use Review Committee of the Kobe University of Medicine (approval number P170705) which complied with the ARRIVE guidelines. GGA (ab146190, Abcam) was dissolved in 100% dimethyl sulfoxide (DMSO) (D8418, Sigma) and stored at − 20 °C.

### Animal grouping and sample collection

For GC–MS analysis, the rats were randomly assigned to 6 groups by lottery method: the sham-operated group (control) and the cerebral ischemia–reperfusion injury model groups, which underwent 1-h MCAO followed by 0, 1, 3, 5, or 24-h of reperfusion (n = 7 in each group; total n = 42). Each rat was euthanized at the designated time point by the intraperitoneal administration of 100–150 mg/kg pentobarbital sodium (Somnopentyl, Kyoritsu Seiyaku Co.) and decapitated after transcardial perfusion with 150–200 mL of ice-cold saline. A total of 30 mg of the infarcted cortex was collected.

For the intracerebroventricular GGA injection study, the rats were randomly assigned to 4 groups by lottery method: the 1-h MCAO and 24-h reperfusion (MCAO-R) after intracerebroventricular GGA injection group (group G), the MCAO-R after intracerebroventricular injection of DMSO as vehicle group (group D), the sham MCAO-R after intracerebroventricular GGA injection group (group GS), and the sham MCAO-R after intracerebroventricular DMSO injection group (group DS). Five rats were used for each group (total n = 20). In addition, six additional rats from group G and D were used to measure the infarct size (total n = 12). Power analysis (two-tailed) with an α of 0.05 and a β of 0.2 (power = 80%) revealed that 6 subjects per group were required to detect a 15% difference in infarct size between group D and group G (estimated standard deviation = 9%). The difference and standard deviation were estimated from the results of our previous study and preliminary study [6]. Age and sex as potential confounders were controlled through inclusion criteria. Decapitation and sample collection were performed in the same manner as above.

### Surgical procedure for MCAO

The rats were anesthetized by the inhalation of 4% isoflurane and maintained with 1.5–2.0% isoflurane in O_2_ (0.4 L/min) and N_2_O (0.8 L/min) using a face mask. Regional cerebral blood flow (rCBF) was measured in the right temporal window by laser doppler flowmetry (TBF-LN1, Unique Medical, Inc.) during surgery. Focal cerebral ischemia was induced by right middle cerebral artery occlusion for 60 min using the suture occlusion technique with 4-0 silicon coated nylon monofilament (Doccol Corporation) with some modifications [5, 30]. Only rats in which the rCBF was reduced by more than 70% of the preischemic value during MCAO were included in subsequent analyses. Rats in which the rCBF was not rapidly restored after thread removal were also excluded from subsequent analyses. Rats with subarachnoid hemorrhage during brain extraction were also excluded.

### Surgical procedure for intracerebroventricular injection

An intracerebroventricular injection of GGA or DMSO was administered 3 h prior to MCAO. We selected a 3 h pretreatment with GGA because our preliminary study demonstrated that phosphorylation of HSP27 was increased 3 h after intracerebroventricular administration of GGA (data not shown). The rats were anesthetized and placed in a stereotaxic apparatus. A small hole was made in the skull 1.0 mm posterior to bregma and 1.8 mm lateral (left) to the midline to puncture the left lateral ventricle. A gas-tight microliter syringe (Hamilton, Reno) was carefully inserted at a depth of 4.5 mm from the skull surface. A total of 10 μL of GGA (0.33 mg, 100 μmol/L) or 10 μL of DMSO was injected at a rate of 2 μL/min. The ventricular puncture was performed to the nonischemic (contralateral to MCAO) side because the puncture itself contributed to the stress-induced local increase of HSP27 (data not shown).

### GC–MS

Low-molecular-weight metabolites were extracted from 30 mg of infarcted cortex, and GC–MS analysis of the infarcted cortex tissue was performed using a GCMS-QP2010 Ultra instrument (Shimadzu Co.) according to previous reports [5, 6]. The MS data were exported in netCDF format. Peak detection and alignment were conducted using MetAlign software (Wageningen UR). The resultant data were analyzed using in-house analytical software (AIoutput) or semiquantitative assessment [31], the peak height of each quantified ion was calculated and normalized to the peak height of 2-isopropylmalic acid as an internal standard.

### RT-PCR

Quantitative RT-PCR analysis of infarcted cortical tissue obtained from each group was performed. Complementary DNA (cDNA) was synthesized from 80 ng of total RNA using a Highcapacity cDNA reverse transcription kit (Thermo Fisher Scientific) according to the manufacturer’s instructions. Quantitative RT-PCR was conducted with 3 μL of diluted cDNA using TaqMan gene expression assays (Applied Biosystems) following the manufacturer’s instructions. β-actin RNA was used as an endogenous control. Quantitative mRNA expression data were acquired and analyzed via the ΔΔCt method. TaqMan gene expression assays with FAM-MGB dye were conducted for the following genes in this study: G6PD (Rn01529640_g1), HSP27 (Rn00583001_g1), and β-actin (Rn00667869_m1). The experiments were repeated two times (each with 2 technical replicates per group).

### Immunoblotting analysis

The protein levels of HSP27, pHSP27, and G6PD in infarcted cortical tissue from each group were determined by immunoblotting according to a previously described method [6]. Five percent bovine serum albumin (BSA, Nacalai Tesque) was used for blocking, and primary antibodies were diluted in Can Get Signal (TOYOBO) at 4 °C overnight. The following antibodies were used: rabbit polyclonal antibodies against G6PD (1:1000, #8866, Cell Signaling Technologies), HSP27 (1:1000, #2442, Cell Signaling Technologies), and pHSP27 (S85) (1:4000, ab5594, Abcam) and a mouse monoclonal antibody against β-actin (1:4000, AM4302, Thermo Fisher Scientific). The blots were quantified using densitometric analysis performed with NIH ImageJ software (ver. 1.51, https://imagej.nih.gov/ij/). β-actin was used as a loading control. The experiments were repeated two times.

### G6PD activity

G6PD activity in infarcted cortical tissue obtained from each group was determined by measuring the rate of production of reduced nicotinamide adenine dinucleotide (NADH) at 450 nm using a Glucose 6 phosphate dehydrogenase assay kit (ab102529, Abcam) according to the manufacturer’s instructions with slight modification [6]. The protein concentration was determined for each sample, and enzyme activity was calculated using a reduced NADH standard curve and are expressed as nmol/min/mg protein. The experiments were repeated three times (each with 2 technical replicates per group)

### Protein carbonyl measurement

Protein carbonylation is an indicator of severe oxidative damage and often leads to the loss of protein function [32]. Protein carbonyl levels in infarcted cortical tissue from each group were measured by using an OxiSelect protein carbonyl ELISA kit (Cell Biolabs) according to the manufacturer’s protocol [6]. The protein concentration was determined for each sample; the level of carbonylated protein was calculated based on a standard curve consisting of a mixture of oxidized and reduced BSA and are expressed as nmol/mg. The experiments were repeated two times (each with 2 technical replicates per group).

### Determination of infarct volume

The infarct volume was measured for group G and D. The rats were euthanized after 24-h reperfusion following 1-h MCAO. The brains were removed after transcardial perfusion and decapitation, and 7 consecutive coronal brain slices (2 mm) from the forebrain were stained with 1% 2, 3, 5-Triphenyltetrazolium chloride (TTC, Sigma) in phosphate buffered saline at 37 °C for 30 min in the dark. Each slice was photographed by a digital camera (GR Digital IV, Ricoh). The infarct area and the ipsilateral hemispheric area were measured directly by the modified autothreshold method using NIH ImageJ software by an observer who was blinded to the treatment [33]. The infarct volume was calculated by multiplying the sum of the areas by the slice thickness (2 mm), and the infarct percentage was calculated as follows: ipsilateral infarct volume/ipsilateral hemisphere volume × 100%.

### Statistical analysis

All statistical analyses were performed with R software version 3.6.1 (https://www.r-project.org). The statistical significance of differences between two groups was determined using the two-sided Mann–Whitney U test. The statistical significance of differences among more than two groups was determined using the Steel–Dwass test. The statistical significance of differences between each experimental mean and the control mean was determined using the nonparametric Dunnett’s test and the Tukey–Kramer test in the nparcomp package in R. Non-parametric statistical tests were used because our data did not follow a normal distribution. A probability (P) value < 0.05 was considered statistically significant.

### Pathway activity profiling analysis

Comparative metabolite changes were determined using PAPi analysis, using an R package [34]. When the correlation between metabolite levels and metabolic pathway activity is analyzed, adequate knowledge of various cellular metabolic pathways is needed, not only for using statistical tools, but mostly for interpreting these data. A new algorithm PAPi that use the metabolite profile and KEGG database, could compare the activity of metabolic pathways between different experimental conditions. The PAPi algorithm was utilized to calculate the activity score for each metabolic pathway. The calculation of activity score is based on the number of metabolites identified from each pathway and their relative abundances, engaging the function buildDatabase to build new local databases which makes use of the information available at KEGG [34]. The local database consists on two CSV files containing the required data about metabolites (Additional file 6: Table S2 [COMPbase]) and metabolic pathways (Additional file 7: Table S3 [PATHbase]). The COMPbase.csv consists of 3 columns, which are names of metabolites, KEGG compound codes and KEGG pathway codes. The PATHbase.csv also consists of three columns, which are names of metabolic pathways, KEGG pathway codes and number of metabolites.

As a result, the activity score represents the likelihood that a metabolic pathway is active inside the cell. Accordingly, it allows the comparison of metabolic pathway activities by use of PAPi functions of performing a t-test or analysis of variance on the activity scores for calculation of p-value. Additionally, PAPi has the function to generate graphical summaries of the results. Before constructing the graph, scaling is performed by setting the activity score of one of the conditions to 0, and by referring to this as the reference condition. Then PAPi scales the activity score of the other target condition relative to the reference condition. This time, only the metabolic pathways with p < 0.05 in analysis of variance test among six different groups, were extracted and shown.

## Supplementary information

**Additional file 1: Table S1.** The pre-processed data of gas chromatography-mass spectrometry.

**Additional file 2: Figure S1.** Full-length blots of β-actin.

**Additional file 3: Figure S2.** Full-length blots of glucose 6-phosphate dehydrogenase.

**Additional file 4: Figure S3.** Full-length blots of heat shock protein 27.

**Additional file 5: Figure S4.** Full-length blots of phosphorylated heat shock protein 27.

**Additional file 6: Table S2.** The information of metabolites to calculate activity scores on pathway activity profiling, whichconsists of the names of metabolites, Kyoto Encyclopedia of Genes and Genomes (KEGG) compound codes and KEGGpathway codes.

**Additional file 7: Table S3.** The information of metabolic pathways to calculate activity scores on pathway activityprofiling, which consists of the names of metabolic pathways, Kyoto Encyclopedia of Genes and Genomes pathwaycodes and number of metabolites.

## Data Availability

The metabolomics data are included in this published article [and its Additional files]. The datasets used and analyzed during the current study except metabolomics data are available from the corresponding author on reasonable request.

## Abbreviations

ROS: Reactive oxygen species
PPP: Pentose phosphate pathway
HSP: Heat shock protein
G6PD: Glucose-6-phosphate dehydrogenase
NADPH: Reduced nicotinamide adenine dinucleotide phosphate
GC-MS: Gas chromatography-mass spectrometry
GGA: Geranylgeranylacetone
PAPi: Pathway activity profiling
KEGG: Kyoto Encyclopedia of Genes and Genomes
NADP: Nicotinamide adenine dinucleotide phosphate
GSSG: Reduce glutathione disulfide
RT-PCR: Real-time polymerase chain reaction
TTC: 1% 2, 3, 5-Triphenyltetrazolium chloride
rCBF: Regional cerebral blood flow
MCAO: Middle cerebral artery occlusion
BSA: Bovine serum albumin
NADH: Nicotinamide adenine dinucleotide
