# Supplementary figures and images for: Geranylgeranylacetone attenuates cerebral ischemia–reperfusion injury in rats through the augmentation of HSP 27 phosphorylation: a preliminary study
Source: BMC Neurosci. 2021 Feb 8;22:9. doi: 10.1186/s12868-021-00614-7 (PMC7869466; doi:10.1186/s12868-021-00614-7)

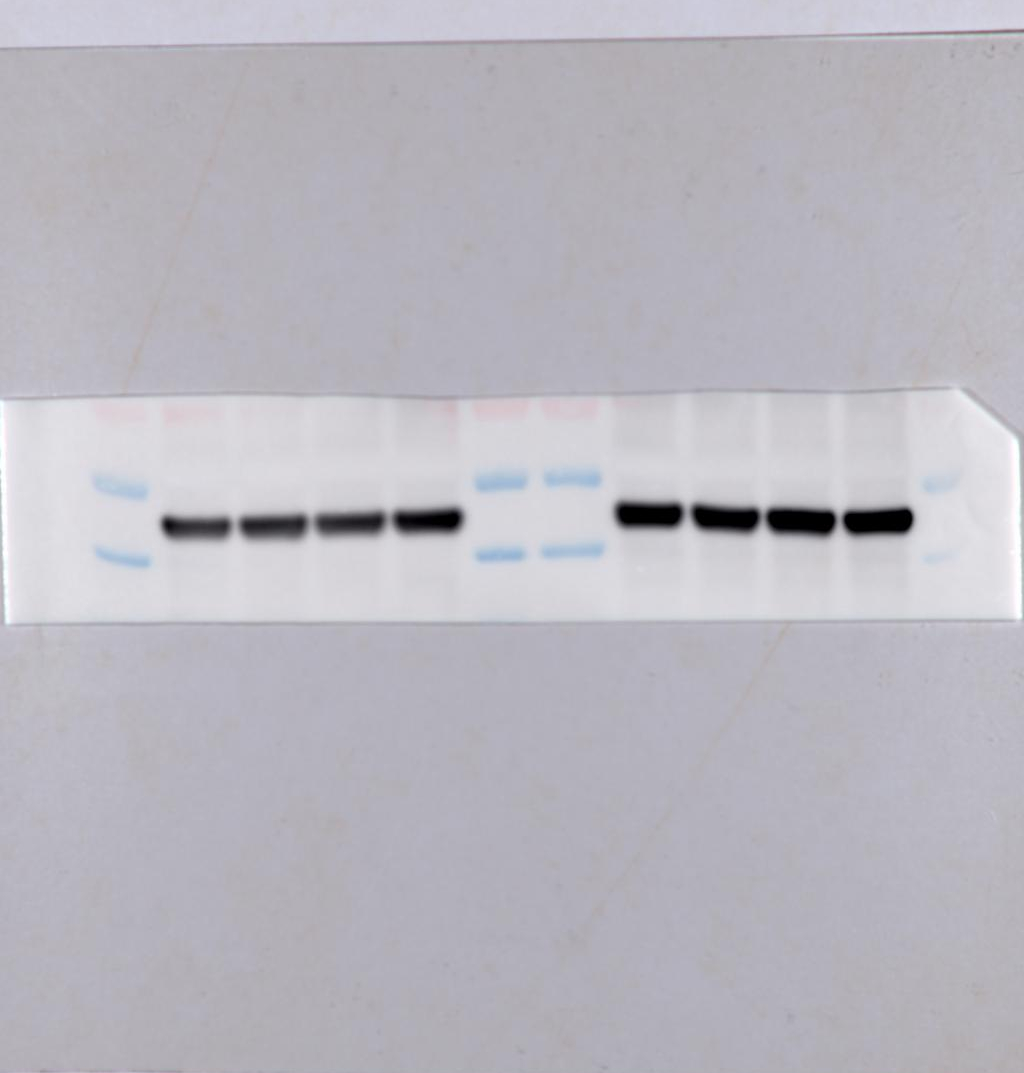

Supplement: Supplementary file 2 — Additional file 2: Figure S1. Full-length blots of β-actin. [file 12868_2021_614_MOESM2_ESM.jpg]

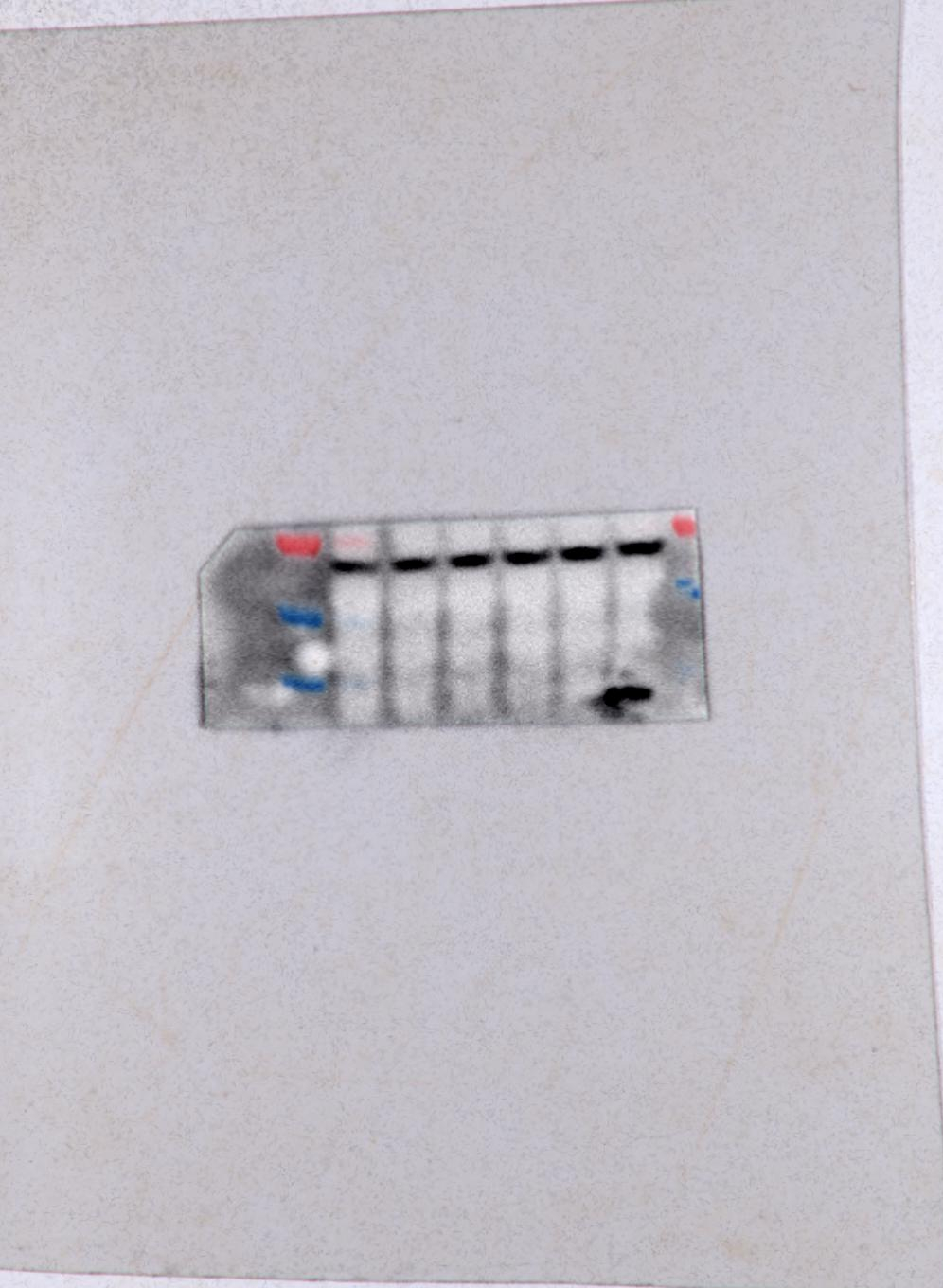

Supplement: Supplementary file 3 — Additional file 3: Figure S2. Full-length blots of glucose 6-phosphate dehydrogenase. [file 12868_2021_614_MOESM3_ESM.jpg]

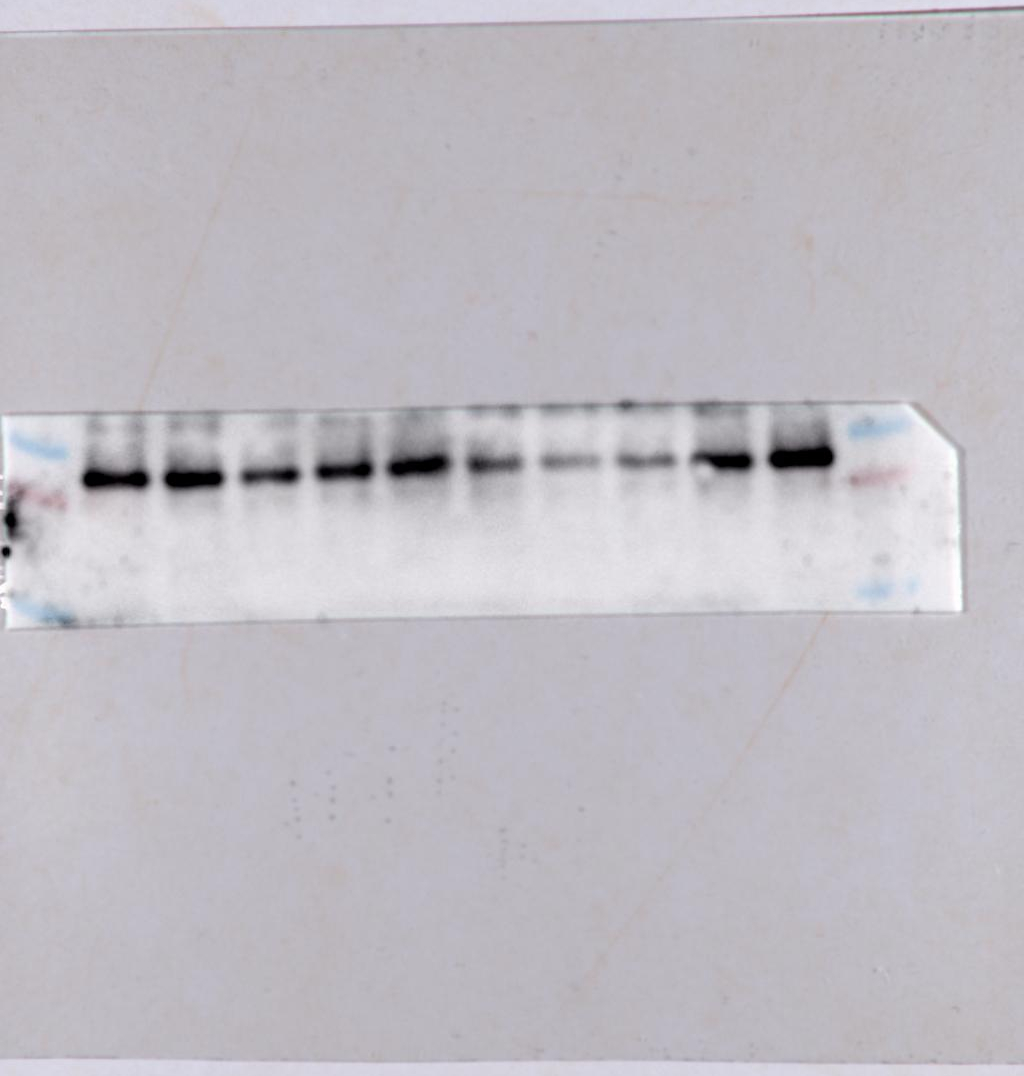

Supplement: Supplementary file 4 — Additional file 4: Figure S3. Full-length blots of heat shock protein 27. [file 12868_2021_614_MOESM4_ESM.jpg]

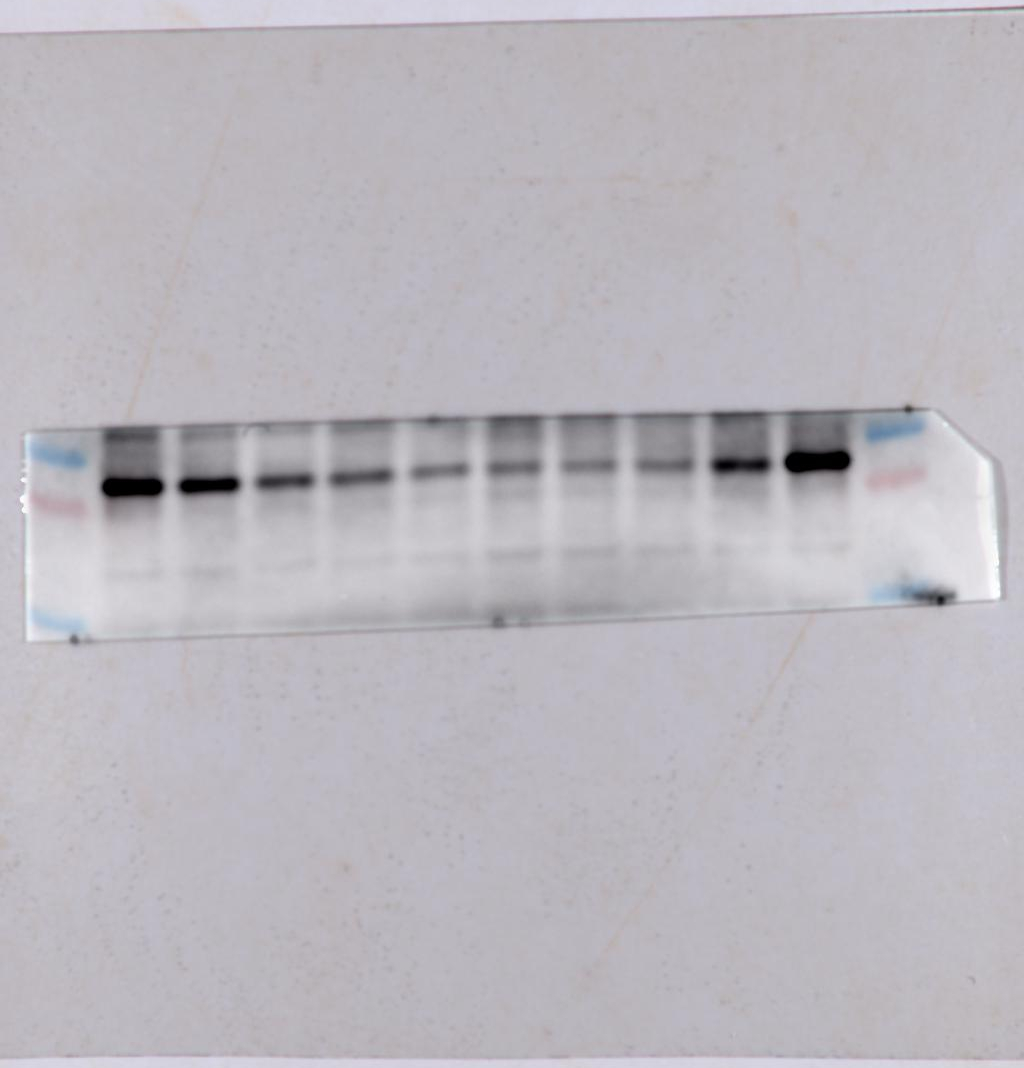

Supplement: Supplementary file 5 — Additional file 5: Figure S4. Full-length blots of phosphorylated heat shock protein 27. [file 12868_2021_614_MOESM5_ESM.jpg]
